# Postmalnutrition weight gain is associated with changes to muscle and energy metabolism in adolescence: a cohort analysis

**DOI:** 10.1016/j.ajcnut.2025.101130

**Published:** 2025-11-29

**Authors:** Elizabeth Wimborne, Amir Kirolos, Natasha Lelijveld, Colleen Deane, Grace O’Donovan, Thandile Nkosi-Gondwe, Amelia Crampin, Marko Kerac, Jonathan R Swann

**Affiliations:** 1 School of Human Development and Health, Faculty of Medicine, University of Southampton, Southampton, United Kingdom; 2 Department of Women and Children’s Health, Institute of Life Course and Medical Sciences, University of Liverpool, Liverpool, United Kingdom; 3 Department of Population Health, Faculty of Epidemiology and Population Health, London School of Hygiene and Tropical Medicine, London, United Kingdom; 4 Emergency Nutrition Network, Kidlington, United Kingdom; 5 Malawi Epidemiology and Intervention Research Unit, Lilongwe, Malawi

**Keywords:** postmalnutrition weight gain, metabolomics, early life, muscle, noncommunicable diseases

## Abstract

**Background:**

Treatment strategies for severe childhood malnutrition often encourage rapid weight gain and catch-up growth. However, the long-term metabolic consequences of such growth are unclear.

**Objectives:**

This study applied metabolomics to investigate how postmalnutrition weight gain (PMWG) in childhood relates to metabolic variation and physiological state in adolescence.

**Methods:**

In an exploratory cohort study, urine and plasma were collected from adolescents (*n* = 151) 15 y after hospitalization for childhood malnutrition in Blantyre, Malawi. Analyses included untargeted urinary ^1^H nuclear magnetic resonance spectroscopy, and targeted plasma liquid chromatography mass spectrometry-based metabolomics and myokine assay. PMWG was assessed using weight-for-age *z*-score (WAZ) at hospital discharge and 1-y follow-up from earlier studies. Adolescent physiology was measured, including muscle function [standing jump length (cm)]. Associations with PMWG were investigated using orthogonal projection to latent structures (OPLS) and regression models.

**Results:**

OPLS demonstrated that a greater increase in WAZ between discharge and 1-y postmalnutrition was associated with distinct plasma and urinary metabolic signatures in adolescence, especially among those with nonedematous malnutrition. This included higher fasting plasma sugars [*β* = 6.40 × 10^3^ μM; 95% confidence interval (CI): 2.99 × 10^3^, 9.81 × 10^3^], triglycerides, phosphatidylcholines, altered amino acids, and lower urinary muscle- and energy-related metabolites. Findings remained significant following adjustment (age, HIV, disability, sex, puberty, socioeconomic status, and minimum admission WAZ). In regression analyses, several of these metabolites positively associated with muscle outcomes, including creatinine (*β* = 13.5 cm; 95% CI: 7.87, 19.2) and β-hydroxy-β-methylbutyrate (*β* = 12.9 cm; 95% CI: 6.97, 18.7) with jump length.

**Conclusions:**

Individuals with greater PMWG exhibited lower muscle-related metabolites and altered energy metabolism in adolescence. It remains unclear whether this reflects inherent differences in how individuals gain weight, or whether early-life weight gain programs future metabolic states. Elucidating these mechanisms will inform interventions to ameliorate long-term health risks, an urgent priority following the growing double burden of malnutrition in low- and middle-income countries.

## Introduction

A rapid health transition is occurring in many African countries where rates of obesity and cardiometabolic diseases are drastically increasing in adulthood, whereas severe childhood malnutrition also remains prevalent [1]. The coexistence of both forms of malnutrition has been termed the “double burden” of malnutrition [2]. Severe malnutrition (SM) includes wasting (low weight-for-height and/or low mid-upper arm circumference) and edematous malnutrition and is often referred to as “severe acute malnutrition” (SAM) [3]. Wasting in childhood remains a major cause of mortality, accounting for over 500,000 deaths annually [4]. At the same time, almost three-quarters of noncommunicable disease (NCD)-related deaths occur in low- and middle-income countries [5,6], demonstrating the overlapping and devastating public health consequences of this phenomenon.

Alongside nutrition transition, lifestyle changes, and urbanization [5,7], the experience of early-life malnutrition has also been linked to elevated cardiometabolic NCD risk [[8], [9], [10]]. A systematic review reported increased cardiovascular disease, metabolic syndrome, and impaired glucose metabolism among childhood malnutrition survivors [8], whereas rapid catch-up growth has been suggested as a risk factor for the development of later-life cardiometabolic disease [11,12]. Few studies, however, have examined the metabolomes of adolescent and adult SM survivors or assessed long-term metabolic consequences of different rates of postmalnutrition weight gain (PMWG). In a Jamaican population, adult SM survivors 20 y post-insult presented with broad metabolic differences compared with community participants [13], whereas lower glucose tolerance and insulin sensitivity were observed in a male Mexican adult population of SM survivors compared with sex-matched controls [14]. A second Jamaican population identified altered lipid metabolism in adult SM survivors [15], with those in the lowest tertile of catch-up presenting more similarly to controls.

Understanding the long-term effects of malnutrition, including the recovery from SM, is becoming increasingly prioritized. It remains unclear whether postnatal SM in childhood presents similar risks surrounding adulthood cardiometabolic disease as that induced by environmental insults in utero. Identifying targets, such as lean muscle mass or potential biomarkers from metabolomic analysis, can provide opportunities for strategic interventions to reduce the public health crisis of the double burden of malnutrition. The “Long-term outcomes after severe childhood malnutrition” (LoSCM) study followed a cohort of children originally hospitalized with SM in Blantyre, Malawi from 2006 to 2007 [[16], [17], [18]]. This study explored the long-term metabolic effects of weight gain after childhood malnutrition into adolescence and the relationship with physiological state. Using untargeted nuclear magnetic resonance (NMR) spectroscopy-based and targeted liquid chromatography mass spectrometry (LC-MS)-based metabolomics, this study characterized lipids and low molecular weight metabolites in urine and plasma from LoSCM adolescents 15 y postdischarge.

## Methods

### Study design

This is an exploratory study investigating the adolescent urinary and plasma metabolomes of a long-term cohort of survivors of severe childhood malnutrition in Malawi 15 y after treatment (LoSCM study) [16]. This cohort has been followed longitudinally since their enrollment in 2006/2007 from the Queen Elizabeth Central Hospital, Blantyre, Malawi. An original 1024 individuals were recruited with a median age of 21.5 mo (IQR: 15–32) into an RCT testing probiotics and prebiotics for SAM [19]. In the initial RCT, SM was defined as weight-for-height 70% median National Center for Health Statistics reference, and/or nutritional edema, and/or a mid-upper arm circumference <11 cm. These were the standard malnutrition program enrollment criteria at that time. This initial RCT showed no overall effect in the primary outcome of nutritional cure (weight-for-height >80% of the National Center for Health Statistics median on 2 consecutive outpatient visits). The secondary outcomes were routine nutrition program performance indicators, and similarly, no clear effect of the intervention was seen. Further follow-ups have occurred at 1- [18] (primary outcome was long-term posttreatment survival; secondary outcome was long-term growth) and 7-y [17,20] (outcomes included anthropometry, body composition, lung function, physical capacity, school achievement, and blood markers of NCD risk) posthospital discharge. For the current LoSCM study, individuals were recontacted by telephone and in person in the community and subsequently assessed at the Queen Elizabeth Central Hospital between 9 September, 2021 and 22 July, 2022. Here, 168 SM survivors were re-recruited. Of the individuals who were not re-recruited to the 15-y follow-up, 11 declined, 10 had died (3 were HIV-positive), and 131 were unable to be contacted due to factors including: no longer residing in the study area, change of phone number, movement to an unknown residence. The sample size of this cohort was therefore constrained by the number of participants originally recruited to the cohort, the number lost to follow-up, and the number consenting to bio-sample collection (Supplemental Figure 1).

Written consent was obtained for all participants, from themselves if aged ≥18 y, or written assent from themselves and written consent from a parent or guardian if aged <18 y. Ethical approval for the study was granted by the Malawi College of Medicine Research and Ethics Committee (reference P.02/21/3269), University of Liverpool Research Ethics Committee (reference number 10126), the London School of Hygiene & Tropical Medicine Research Ethics Committee (reference number 26299), and the University of Southampton Research Ethics Committee (reference number 80095.A1). Additionally, the study was prospectively registered in the International Standard Randomized Controlled Trial Number Registry (ISRCTN17238083). This manuscript followed Strengthening the reporting of observational studies in epidemiology (STROBE) cohort reporting guidelines [21].

### Participant data and anthropometry collection

During the original studies, weight (Tanita 1582 digital scales) and length (height boards) were measured at hospital admission, discharge, and the 1-y postdischarge follow-up [18,19]. Demographic data were collected from the participants during the follow-up assessment. This included HIV status, puberty status (prepubertal assigned if individuals self-reported a score of ≤2 in both genitalia and pubic hair Tanner staging questions), and presence of disability [determined across several domains using the Washington Group disability questions [22] with a score of 3 (“a lot of difficulty”) or 4 (“unable to do”) in any domain]. Malawi 2015 Demographic and Health Survey questions were used to assess socioeconomic status, from which a score was determined for each participant using principal component analysis. Additionally, anthropometric (height, weight, and body composition) and muscle strength measures (standing long jump and hand-grip strength) were captured independently by 2 study nurses who had received updated WHO anthropometry training. Measures were repeated if a variation greater than the prespecified limits was detected. These included height (height board; SECA 213, SECA), weight (digital scales; SECA 877, SECA), body composition [BIA; Quadscan 4000 device (Bodystat)], standing long jump (tape measure from set mark to heel), and hand-grip strength (Takei Grip-D device). Full details of collection have been published previously [16].

Venous blood and urine were sampled from all willing participants during the morning. Venous blood was collected via a cannula into a lithium heparin tube and spun to collect plasma within 15 min of extraction. Urine was collected as a spot sample into a collection pot. Plasma and urine samples were stored at –80°C for shipping to the United Kingdom for metabolomic analysis. Participants were requested to fast overnight for ∼10 h before sample collection, which was confirmed by self-report during the assessment. For plasma analyses, only samples from fasted individuals were analyzed to minimize the acute effects of feeding. For urine, samples from both fasted and nonfasted participants were included, as urinary metabolites are less directly influenced by fasting status. However, fasting was included as a covariate in statistical analysis. Urinary metabolic profiles were generated for all participants with an available sample (*n* = 151), whereas plasma profiles were generated for a subset of participants (*n* = 96 metabolites*/n* = 67 myokines).

Oral glucose tolerance tests were performed on a subset of participants (those who confirmed fasting, consented to multiple blood draws and arrived with sufficient time for the test to be completed). These participants consumed 75 mg glucose in a 300 mL Rapilose OGTT solution (Penlan Healthcare), with repeated venous blood collection after 30, 60, 90, and 120 min.

A lean mass index was calculated (1/impedance). Following the OGTT HOMA-IR, the Matsuda index, insulinogenic index and disposition index were calculated, with full details previously published [16]. The triglyceride (TG)–glucose index has been reported as a reliable alternative measure of insulin resistance [23]. Fasting plasma hexose was measured during the metabolomics in this cohort. Therefore, a surrogate “TG-hexose” index was also calculated in this population using the formula ln(sum fasting TG ∗ fasting hexose)/2.

### ^1^H NMR spectroscopy

Urinary metabolic profiles were measured by ^1^H NMR spectroscopy. Urine (540 mL) was combined with 60 mL of potassium buffer solution (pH 7.4, 100% D_2_O) containing 1 mM of the internal standard, 3-trimethylsilyl-1-[2,2,3,3-2H4] propionate (TSP). Samples were vortexed to mix, spun at 10,000 g, and 575 mL of the supernatant was transferred to 5 mm NMR tubes. A pooled sample was prepared to serve as a biological quality control and analyzed repeatedly throughout the run to evaluate reproducibility.

All samples were measured with a 700 MHz Bruker NMR spectrometer equipped with a cryoprobe and refrigerated SampleJet autosampler maintained at 6°C (Bruker Biospin GmbH). A standard 1-dimensional solvent suppression pulse sequence (relaxation delay, 90° pulse, 4-ms delay, 90° pulse, mixing time, 90° pulse, acquire free induction decay) was used to measure each sample. Each spectrum was acquired with 32 scans, 4 dummy scans, 64,000 frequency domain points and a spectral window set to 20 ppm (parts per million). All spectra were automatically phase and baseline corrected and referenced to the TSP resonance at δ 0.0 in Topspin 3.2 (Bruker Biospin GmbH). The raw spectra were digitized, aligned, and normalized in Matlab (version 2022b, MathWorks Inc.) using the Imperial Metabolic Profiling and Chemometrics Toolbox (https://github.com/csmsoftware/IMPaCTS). Redundant spectral peaks (TSP, water, and urea) were excised, and the resulting spectra were manually aligned using a recursive segmentwise peak alignment method. A probabilistic quotient normalization method was used to reduce the effects of variation in sample dilution. Single representative peaks from the annotated metabolites were integrated, log-transformed and used for further analysis.

### Liquid chromatography mass spectrometry

Plasma metabolic profiles were measured on a Waters Acquity Premier ultraperformance liquid chromatography (UPLC) system coupled to a Xevo TQ-XS mass spectrometer using the Biocrates MxP Quant 500 kit (Biocrates Life Sciences) [24]. This method used LC-MS for the analysis of small molecules and flow injection analysis (FIA) for the lipids and hexoses. This was acquired following the manufacturer’s instructions. Quantification was achieved using a 7-point standard curve. Briefly, 10 μL of thawed samples were added to a 96-well plate containing inserts of internal standards supplied by Biocrates and dried using nitrogen flow. Samples were incubated with 5% phenyl isothiocyanate, dried, shaken following the addition of 5 mM ammonium acetate in methanol, before being transferred to separate 96-well plates diluted with either pure water or FIA solvent. Metabolites were measured in positive and negative ionization modes. The LC-MS method involved the injection of 5 μL of extract into the Biocrates MxP Quant 500 kit UPLC column at 50°C using a 5.8 min solvent gradient with 0.2% formic acid in water and 0.2% formic acid in acetonitrile. Second, the FIA–MS/MS method involved the injection of 20 μL of extracts. The raw data from the UPLC-MS and FIA analyses were processed using the Biocrates webIDQ software in accordance with the manufacturer’s protocol. To minimize technical variation across the different plates, batch correction was performed using standard quality control samples included throughout each plate. The lower limit of detection (LOD) for each metabolite was calculated as a 3-fold increase from the noise in the blank sample. Metabolites with missing values (<LOD) in >80% of samples, and/or >15% coefficient of variation (CV) in the internal standards in QC samples were excluded from the overall dataset (Supplemental Table 1). A total of 204 metabolites were retained. Missing values in these metabolites were replaced with the LOD/√2 for that metabolite. Metabolite values above the upper limit of quantification (ULOQ) were replaced with the upper limit of quantification.

### Myokines

Plasma myokine profiles were measured using the Milliplex human myokine magnetic bead panel (cat# HMYOMAG-56K, Millipore) following instructions from the manufacturer [25]. These kits were used to quantify: *1*) apelin, *2*) fractalkine, *3*) brain-derived neurotrophic factor (BDNF), *4*) erythropoietin (EPO), *5*) osteonectin, *6*) leukemia inhibitory factor (LIF), *7*) IL-15, *8*) myostatin, *9*) fatty acid-binding protein 3 (FABP3), *10*) irisin, *11*) follistatin-related protein 1 (FSTL1), *12*) oncostatin M (OSM), *13*) IL-6, *14*) fibroblast growth factor 21 (FGF21), and *15*) musclin.

Plasma samples were vortexed, centrifuged, and then diluted in a 2:1 ratio of assay buffer to sample. Antibody-immobilized beads were prepared by sonicating each antibody bead vial for 30 s and vortexing for 1 min. From each vial, 150 mL was combined with 0.75 mL of bead dilutant. Quality controls were reconstituted by adding 250 mL of deionized water, followed by vortexing to ensure thorough mixing. Standards, quality controls, 75 plasma samples, and 1 pooled sample were prepared on a 96-well plate. Standards and quality controls were analyzed in duplicate. Each well received 200 mL of wash buffer, was sealed, and shaken at 500 × *g* for 10 min before decanting. Then, 25 mL of standards, controls, samples, assay buffer, serum matrix, and antibody beads were added to the appropriate wells. The plate was sealed, wrapped in foil, and incubated at 2–8°C for 18 h with shaking. After incubation, a handheld magnet retained the beads while 3 wash steps were performed. Detection antibodies (25 mL) were then added to each well, and the plate was incubated at room temperature for 1 h with shaking. Next, 25 μL of Streptavidin-Phycoerythrin was added to each well without aspiration, followed by a final 30-min incubation in the same conditions as above. After washing, 150 μL of sheath fluid PLUS was added to all wells, and the beads were resuspended by shaking at room temperature for 5 min.

The plate was run on a Bio-Plex 200 instrument using Bio-Plex Manager 6.1 Security Edition Software. Validation and calibration plates were analyzed before running the multiplex plates. Standard curves and resultant concentrations were generated for each myokine by the Bio-Plex Manager software. Samples were checked for a bead count of >50 and myokines with >80% missing values, and/or >15% CV in the duplicated QC samples were removed from future analysis (Supplemental Table 2). To account for technical variation between the 2 plates, plate number was included as a covariate in the downstream analysis. Missing values under the LOD were imputed with the LOD/√2, and values above the ULOQ were replaced with the ULOQ. Of the 15 myokines measured, 7 were detected consistently, which included BDNF, EPO, FABP3, FSTL-1, oncostatin M, FGF21, and osteonectin.

### Exposures and outcomes

The primary exposure explored in this study was PMWG, defined as the change in weight-for-age *z*-score (ΔWAZ) between hospital discharge and the 1-y follow-up and during hospitalization, with stratification by edema status at admission. Outcomes included urinary and plasma metabolomic profiles, circulating myokines, muscle function and composition (lean mass index, jump length, grip strength), and derived indices of insulin sensitivity and resistance (including the TG-hexose index). Additional analyses examined associations between metabolomic features and other outcomes (muscle phenotype, myokines, TG-hexose index).

### Statistical analysis and data visualization

**FIGURE 1:**
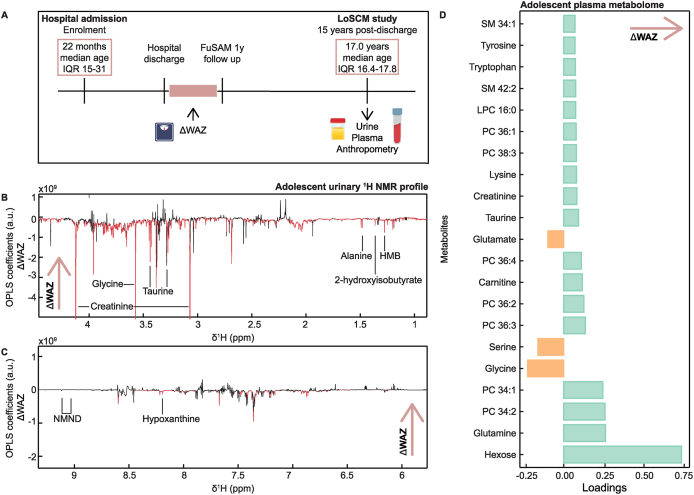
Postmalnutrition weight gain associated with altered adolescent urinary metabolome. (A) Timeline of sample collection and anthropometric measurement from the LoSCM study. (B–C) Associations between the ^1^H nuclear magnetic resonance (NMR) urinary metabolic profile and change in weight-for-age *z*-score (ΔWAZ) between hospital discharge and the 1-y follow-up based on an orthogonal projection to latent structures (OPLS) model. OPLS model generated in adolescent SM survivors who presented without edema during hospital admission (*n* = 28). Subsets of the full spectra including the regions δ0.9–4.2 and δ6.7–10.0 are displayed. Each plot displays the coefficient of the OPLS model with intensity in arbitrary units. Negative coefficients with increased weight gain point downward, and positive coefficients point upward. Statistically significant peaks from the OPLS models highlighted in red. (D) Bar chart of loadings between plasma metabolites identified in OPLS models with ΔWAZ between hospital discharge for childhood SM and the 1y follow-up for those who presented without edema during hospital admission (*n* = 28). FuSAM, follow-up of postdischarge growth and mortality after treatment for severe acute malnutrition; HMB, β-hydroxy-β-methylbutyrate; LoSCM, long-term outcomes after severe childhood malnutrition in adolescents in Malawi; LCP, lysophosphatidylcholine; NMND, *N*-methylnicotinamide; ppm, part per million; PC, phosphatidylcholine; SM, sphingomyelin.

Orthogonal projections to latent structures (OPLS) models were constructed using the ^1^H NMR spectra of the urine or the plasma metabolic profiles of the SM survivors, investigating metabolic features associated with anthropometric growth during or recovery from hospitalization (Figure 1A). For each model, the X matrix was composed of the respective ^1^H NMR spectra, plasma metabolites, or myokine concentrations, whereas the variable of interest was used as the predictive Y component (ΔWAZ hospital admission to discharge, ΔWAZ hospital discharge to 1-y follow-up, and presence of edema during hospitalization). A 7-fold cross-validation approach was used to assess the predictive capacity of each model, and permutation testing evaluated the model validity (1000 permutations; *P* < 0.05 were considered significant). Model performance was summarized by the *Q*^2^ statistic, which quantifies the model's predictive ability based on cross-validated prediction of the outcome variable. Variable importance was assessed using the variable importance in projection (VIP) score, with features exhibiting a VIP >1 considered important and retained for further analysis. Loadings were examined to describe the direction and magnitude of association of individual features with the outcome. The OPLS models of the urinary ^1^H NMR spectra were created using in-house scripts in Matlab (version R2022b, MathWorks Inc.). The OPLS models of the plasma metabolome and myokine profiles following Pareto scaling were created in R (version 4.5.0) using the ropls package [26].

Linear regression models were fitted using the lm() function in R (base *stats* package), to examine associations of PMWG (ΔWAZ during the first-year postdischarge), muscle function, the TG-to-hexose (TG-hex) ratio, indices of insulin sensitivity and resistance, and circulating myokines with urine and plasma metabolites identified from multivariate OPLS models, as well as with one another. Models were performed unadjusted and adjusted for age (y), HIV status (positive/negative), disability (present/absent, defined by Washington Group criteria), sex (male/female), prepubertal status (prepubertal compared with pubertal, based on Tanner staging), socioeconomic status (principal components analysis-derived score), the minimum WAZ during admission, and fasting (yes/no, urine only) unless specified. Missing values in covariates were handled using complete-case analysis. Scatter plots were created using covariate-adjusted values. These were obtained as residuals from regression models where each feature was regressed on these covariates. A Benjamini–Hochberg false discovery rate (FDR) correction for multiple testing was applied, and features with a corrected *P* < 0.05 were statistically significant in the analysis.

Analyses were performed including all SM survivors and stratified by the presence of edema during hospitalization. Myokines were only measured in 7 SM survivors who had not experienced edema during hospitalization; therefore, analysis of this assay was not stratified by the presence of edema. However, edema presentation was included as a covariate in this analysis.

## Results

### Participants

In total, urinary metabolic profiles were generated for 151 adolescent SM survivors (Supplemental Figure 1). Plasma metabolic and myokine profiles were also acquired for a subset of these participants (metabolites *n =* 96; myokines *n* = 67). The characteristics of these individuals are detailed in Supplemental Table 3. The median age of SM survivors was 17.0 y, and 45.5% were female. Of these, 53 (34.9%) had a positive HIV status, 12 (7.9%) presented with a disability, and 5 (3.3%) were prepubertal. During hospitalization for childhood malnutrition, 123 (80.9%) presented with edema. The mean change in WAZ during hospitalization was + 0.03 (IQR: 0.01–0.05), whereas the mean ΔWAZ from hospital discharge to the 1-y follow-up was +0.05 (IQR: 0.00–0.09). The mean duration between hospital discharge and the 1-y follow-up was 16.0 mo [95% confidence interval (CI): 12.1, 16.6]. Of the SM survivors, 12 had missing values for change in WAZ (ΔWAZ) during hospitalization, 33 had missing values for ΔWAZ from hospital discharge to the 1 y follow-up.

### Urinary markers of muscle metabolism were negatively associated with early-life PMWG

Change in WAZ between hospital discharge and the 1-y follow-up in adolescent SM survivors (ΔWAZ) was used as a measure of PMWG. A significant OPLS model with modest predictive power (*Q*^2^) was obtained, indicating that PMWG was associated with variation in the adolescent urinary metabolome (*Q*^2^ = 0.008; *P* = 0.04, *n* = 151). From this model, adolescents with greater PMWG in early life had lower urinary creatinine (*β* = –2.41; 95% CI: –4.42, –0.41), 2-hydroxisobutyrate (*β* = –3.09; 95% CI: –5.48, –0.71), and pyruvate (*β* = –2.51; 95% CI: –4.46, –0.56). These relationships remained significant in linear regression models adjusted for sex, age, socioeconomic status, minimum WAZ during hospital admission, and fasting, with the results displayed in Supplemental Table 4.

When stratified by the presence of edema during hospitalization, a significant OPLS model was only observed for those who presented without edema during hospitalization, with a substantial improvement in predictive capacity (no edema *Q*^2^ = 0.32, *P* = 0.01, *n* = 28, Figure 1B–C). Here, individuals with greater ΔWAZ in the first-year postdischarge had lower urinary β-hydroxy-hydroxymethylbutyrate (HMB), alanine, creatinine, taurine, glycine, 2-hydroxisobutyrate, hypoxanthine, 4-hydroxyphenylacetate, and *N*-methylnicotinamide (NMND). Such negative relationships between ΔWAZ and HMB (*β* = –5.28; 95% CI: –9.60, –0.96), creatinine (*β* = –5.84; 95% CI: –9.51, –2.16), 2-hydroxisobutyrate (*β* = –5.62; 95% CI: –9.54, –1.70), hypoxanthine (*β* = –6.00; 95% CI: –9.87, –2.13), 4-hydroxyphenylacetate (*β* = –5.15; 95% CI: –9.33, –0.96), and NMND (*β* = –5.80; 95% CI: –9.81, –1.80) remained significant in linear regression models adjusting for sex, age, socioeconomic status, minimum WAZ during hospital admission, and fasting, with the results displayed in Supplemental Table 5.

### Plasma metabolome variation associated with greater postmalnutrition recovery growth in those with no edema at hospital admission

To investigate relationships between PMWG in childhood and the adolescent systemic metabolome, plasma metabolic profiles were measured in a subset of fasted adolescent SM survivors (*n* = 96) using a semitargeted LC-MS profiling method. A significant OPLS model was observed, indicating that childhood PMWG was associated with variation in the adolescent plasma metabolome when only considering SM survivors who presented without edema during hospital admission (*Q*^2^ = 0.23; *P* = 0.03; *n* = 28; Figure 1D). Here, ΔWAZ in the first-year postdischarge positively associated (VIP >1) with 18 circulating metabolites comprising plasma hexose including glucose, 5 amino acids (glutamine, lysine, taurine, tryptophan, tyrosine), 7 phosphatidylcholines, carnitine, creatinine, 2 sphingomyelins (SM: 34:1, SM: 42:2), and 1 lysophosphatidylcholines (LPC: 16:0). Conversely, ΔWAZ was negatively associated with the circulating abundance of 3 amino acids (glycine, serine, and glutamate).

**FIGURE 2:**
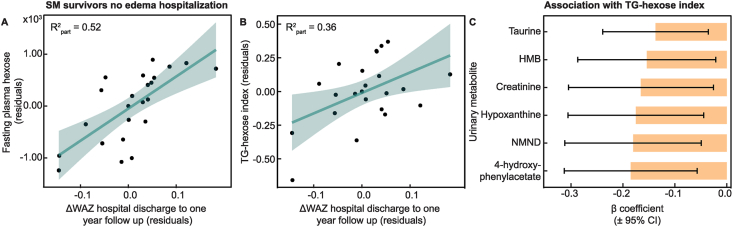
Postmalnutrition weight gain in childhood associated with increased fasting plasma sugars in adolescence. (A) Scatter plot of change in weight-for-age *z*-score (ΔWAZ) in the first-year postdischarge (PMWG) against fasting plasma hexose in adolescent SM survivors of nonedematous childhood malnutrition (*n* = 28). (B) Scatter plot of PMWG against triglyceride (TG)-hexose index in adolescent SM survivors of nonedematous childhood malnutrition (*n* = 28). (C) Bar chart of β coefficient of adjusted linear regression between urinary metabolites associated with PMWG and total TG-hexose index in adolescent SM survivors of nonedema childhood malnutrition (*n* = 28). Bars represent effect size, and horizontal lines indicate 95% confidence intervals (CIs). Points represent covariate-adjusted values (residuals after adjustment for sex, socioeconomic status, age, HIV status, disability, minimum enrollment WAZ, and prepubertal status). Partial *R*^*2*^ values indicate the additional variance explained by each metabolite after covariate adjustment. HMB, β-hydroxy-β-methylbutyrate; NMND, *N*-methylnicotinamide; SM, severe malnutrition (weight-for-height <70% median National Center for Health Statistics reference, and/or nutritional edema, and/or a mid-upper arm circumference <11 cm).

When investigated using regression models, ΔWAZ in the first-year postdischarge remained positively associated with the circulating abundance of hexose (Figure 2A; *β* = 6.40 × 10^3^ μM; 95% CI: 2.99 × 10^3^, 9.81 × 10^3^), 7 phosphatidylcholines, carnitine (*β* = 7.14 × 10^2^ μM; 95% CI: 2.68 × 10^2^, 1.16 × 10^3^), and LPC 16:0 (*β* = 6.73 × 10^2^ μM; 95% CI: 1.47, 1.33 × 10^3^) after adjustment for covariates (minimum enrollment WAZ, sex, socioeconomic status, age, HIV status, disability and prepubertal status; Supplemental Table 6). No significant OPLS models were observed for those who presented with edema at hospital admission (*n* = 68), or when the whole adolescent SM survivor population was included (*n* = 96).

Neither the plasma nor urinary metabolomes (of the whole or nonedematous SM survivor groups) were associated with PMWG at other time points (i.e. during hospitalization), nor with the clinical phenotype of SM (i.e. presence of edema at admission).

### Plasma sugar–TG index associated with PMWG

Following the strong, positive association between plasma hexose and PMWG in the nonedematous adolescent SM survivor population (Figure 2A), linear regression models were generated to investigate associations between the TG-hexose index and PMWG, plasma and urinary metabolites, and muscle phenotype. Positive relationships were observed between ΔWAZ in the first-year postdischarge and the TG-hexose index after adjustment for covariates in those who did not experience edema during hospitalization [*β* = 1.84; 95% CI: 0.65, 3.04 (Figure 2B)], and the whole adolescent SM survivor population (*β* = 1.04; 95% CI: 0.31, 1.77). A trend was noted for those who experienced edema (*P* = 0.09).

Additionally, significant negative relationships were observed between the urinary metabolites taurine (*β* = –0.14; 95% CI: –0.24, –0.036), hypoxanthine (*β* = –0.18; 95% CI: –0.31, –0.045), 4-hydroxyphenylacetate (*β* = –0.18; 95% CI: –0.31, –0.057), HMB (*β* = –0.15; 95% CI: –0.29, –0.021), NMND (*β* = –0.18; 95% CI: –0.31, –0.050), and creatinine (*β* = –0.17; 95% CI: –0.31, –0.026) and the TG-hexose index in the nonedema population after adjustment for covariates and multiple testing (Figure 2C; Supplemental Table 7). These associations were not significant when the whole SM population was considered.

Interestingly, this marker did not associate with other measures of insulin sensitivity/resistance calculated in this population (HOMA-IR, disposition index, insulinogenic index, Matsuda index; Supplemental Table 8). Similarly, no significant associations were observed between such measures of insulin sensitivity/resistance and the urinary/plasma metabolic changes, other phenotypic measures (lean muscle mass, jump length, grip strength), or PMWG.

### PMWG negatively associated with muscle-related metabolites and muscle factors

As PMWG was negatively associated with several metabolites related to muscle metabolism, their association with functional and compositional muscle was explored. Adjusted linear regression models were created between the relative abundance of urinary metabolites or systemic concentrations of plasma metabolites and measures of muscle function (lean muscle, standing jump length, grip strength).

**FIGURE 3:**
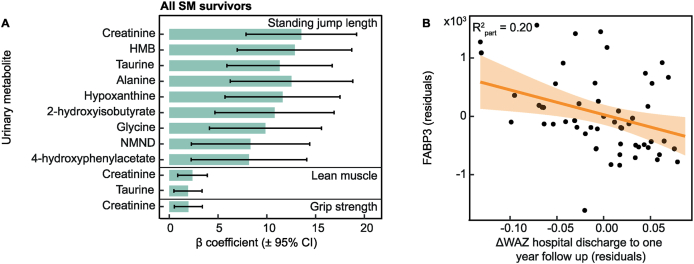
Postmalnutrition weight gain in childhood associated with decreased muscle-related metabolites in adolescence. (A) Bar chart of β coefficient of adjusted linear regression between urinary metabolites and measures of muscle function in adolescent SM survivors (*n* = 151). Bars represent β coefficient, and horizontal lines indicate 95% confidence intervals (CIs). (B) Scatter plot of fasted plasma FABP3 and change in weight-for-age *z*-score (ΔWAZ) in the first-year postdischarge in a subset of the adolescent SM survivors with myokine measures (*n* = 67). Points represent covariate-adjusted values (residual adjustment for minimum enrollment WAZ, sex, socioeconomic status, age, HIV status, disability, and prepubertal status). Partial *R*^*2*^ values indicate the additional variance explained by each metabolite after covariate adjustment. FABP3, fatty acid-binding protein 3; HMB, β-hydroxy-β-methylbutyrate; NMND, *N*-methylnicotinamide; SM, severe malnutrition (weight-for-height <70% median National Center for Health Statistics reference, and/or nutritional edema, and/or a mid-upper arm circumference <11 cm).

Considering all SM survivors, urinary HMB (*β* = 12.9 cm; 95% CI: 6.97, 18.7), creatinine (*β* = 13.5 cm; 95% CI: 7.87, 19.2), alanine (*β* = 12.5 cm; 95% CI: 6.26, 18.8), hypoxanthine (*β* = 11.6 cm; 95% CI: 5.71, 17.5), 2-hydroxyisobutyrate (*β* = 10.8 cm; 95% CI: 4.70, 16.9), taurine (*β* = 11.3 cm; 95% CI: 5.90, 16.7), NMND (*β* = 8.33 cm; 95% CI: 2.28, 14.4), 4-hydroxyphenylacetate (*β* = 8.17 cm; 95% CI: 2.24, 14.1), and glycine (*β* = 9.86 cm; 95% CI: 4.12, 15.6) were positively associated with standing jump length (Figure 3A; Supplemental Table 9). Additionally, urinary creatinine (*β* = 2.39 cm^2^/Ω; 95% CI: 0.87, 3.90) and taurine (*β* = 1.92 cm^2^/Ω; 95% CI: 0.47, 3.37) positively associated with lean muscle mass (Figure 3A), and creatinine with grip strength (*β* = 1.97 kg; 95% CI: 0.53, 3.40; Figure 3A). No associations were significant after correcting for multiple testing when considering only the adolescent SM survivors who did not experience edema in the hospital.

To further investigate the relationship between this altered muscle-related metabolite profile and muscle function, several myokines were measured in fasted plasma samples of a subset of adolescent SM survivors (*n* = 67) using a magnetic Milliplex assay. Adjusted linear regression models were created to explore associations between circulating myokines and PMWG (Supplemental Table 10). A negative trend was observed between ΔWAZ in the first-year postdischarge and FABP3; however, this relationship did not reach statistical significance following correction for multiple testing [(*P* = 0.01, FDR *P* = 0.1; *β* = –4256.12; 95% CI: –7690, –818); Figure 3B]. Multivariate OPLS models were also generated to investigate whether PMWG was represented in the global myokine profile; however, no significant models were observed. Additionally, no associations were observed between FABP3 and measures of muscle function or the identified urinary and plasma metabolites.

## Discussion

This exploration of the metabolic patterns in survivors of early-life malnutrition adds important new information to our previous description of clinical and body composition phenotypes [16]. Whereas there were few clinically apparent differences between malnutrition survivors and sibling/community controls [16], the rate of weight gain in childhood following hospital treatment for SM was associated with distinct urinary and plasma metabolomic profiles 15 y later. This was most pronounced in those who experienced nonedematous malnutrition. Specifically, adolescent SM survivors who experienced the greatest ΔWAZ in the first-year postdischarge had greater fasting plasma hexoses (sugars), TGs, phosphatidylcholines, altered abundances of amino acids, and lower cholesterol esters, as well as lower urinary metabolites related to muscle mass and metabolism, which in turn were associated with muscle function. Additionally, there was a strong positive association between PMWG and the TG-hexose index, a proxy for a TG–glucose index, identified as a biomarker for insulin resistance [23]. Such metabolic variation may reflect an elevated risk of future cardiometabolic disease in this population.

Early-life SM has been associated with increased later-life cardiometabolic disease risk [[9], [10], [11]], with excess catch-up growth following PMWG implicated as a risk factor [11,12,15,27]. Elevated fasting plasma sugars and TGs, hallmark features of the metabolic syndrome [28], may reflect insufficient insulin secretion or tissue insulin resistance following PMWG. Consistent with our results, other work has also demonstrated a greater risk for insulin resistance in individuals who experienced nonedematous malnutrition [29]; however, this has not been associated with their rate of PMWG. Moreover, the TG–glucose index, also associated with PMWG here, has successfully predicted the risk of developing type-2 diabetes, cardiovascular events, and metabolic syndrome [[30], [31], [32]]. Similarly, plasma, PC: 36:1, PC: 36:3, and PC: 38:3, were increased with PMWG and have been linked to coronary artery disease [33], whereas 5 of the identified phosphatidylcholines were elevated in a murine model of atherosclerosis [34].

Lean muscle is an established marker for metabolic health and inversely related to cardiometabolic risk [[35], [36], [37]]. In this population, PMWG is inversely associated with the urinary abundance of several muscle-related metabolites, which in turn are related to muscle function. These included creatinine, a common urinary marker of muscle mass, and HMB, a breakdown product of leucine. HMB both enhances muscle protein synthesis via mechanistic target of rapamycin (mTOR) signaling and reduces muscle protein breakdown via suppression of proteolytic pathways, including the ubiquitin-proteasome and autophagy-lysosome systems [38,39]. It has also been associated with improved cardiometabolic outcomes, including enhanced glucose tolerance and insulin sensitivity in murine models [40] and lipid metabolism in humans [40]. FABP3, a myokine that facilitates fatty acid uptake and utilization in muscle [41], was inversely associated with PMWG. FABP3-deficient mice have dampened fatty acid uptake and reduced exercise capacity, alongside elevated glucose consumption [42]. This is consistent with our previous finding that FABP3 expression was downregulated with disuse-induced muscle atrophy [43], and together with these metabolomic and muscle phenotype associations, supports the interpretation that PMWG may leave lasting alterations in muscle metabolic capacity.

These findings highlight that greater PMWG in early life, especially following nonedematous malnutrition, is linked to metabolic and muscle alterations in adolescence, suggesting increased future cardiometabolic risk. Whether these metabolic changes reflect an inherent predisposition to store fat at the expense of muscle (i.e. PMWG as a biomarker of vulnerability) or arise from metabolic programming driven by greater PMWG in childhood remains unclear. Nonedematous malnutrition has been linked to low birth weight (333–500 g lower birth weights reported than those with edematous malnutrition) [44,45], as well as metabolic changes in later life [44], demonstrating the potential contributions of prenatal factors to long-term disease risk. If PMWG reflects an inherent vulnerability, it is not an appropriate marker for nutritional rehabilitation, but could be measured to tailor nutritional, lifestyle, and physical activity programs to reduce NCD risk in susceptible individuals across the life course. Conversely, if the PMWG itself was the driver of such metabolic changes, nutritional rehabilitation strategies should be refined to promote healthier patterns of weight gain, for example, by prescribing food rations at the lower end of the recommended energy/protein range rather than at the upper end as currently happens in most treatment programs. This might result in slower, steadier, and potentially healthier weight gain. Regardless, this study has identified a high-risk population who require monitoring and targeted interventions to reduce future public health burden.

There are several research and clinical implications of this study. First, future research should examine whether similar associations are seen in other malnutrition or famine survivor cohorts, and assess if the metabolomic markers identified here predict later NCD outcomes. Assuming so, this work creates important opportunities to refine and improve treatments for SM. An immediate potential clinical implication is that program managers and supervisors should not focus quite so much on *program*-level weight gain as some currently do [46]. Not only is this not a critical outcome, % cure; % death and postmalnutrition development are far more important, and weight gain is an imperfect proxy of these [47], but this work shows that too rapid weight gain may have adverse longer-term implications. Long-term follow-up is rarely possible, so treatment implications remain unknown. Hence, if our biomarkers of risk are indeed valid, measuring them in intervention trials could give early insight into the long-term risks and benefits. Additionally, the use of physical therapy to promote lean rather than fat mass during this weight gain period also warrants investigation in longitudinal cohorts.

Strengths of this study include the 15-y longitudinal characterization of participants, enabling exploration of novel biological questions. Metabolomic profiling complements previous clinical data [16] and may offer early insights into cardiometabolic disease risk. Limitations include the cohort’s survivor bias due to the high mortality observed over the 15 y [17,18]. Therefore, only metabolic profiles of the “fittest” survivors were explored, possibly obscuring patterns linked to higher vulnerability. It should also be noted that the weight gain considered is during the year postdischarge. Although the participants were not discharged into an obesogenic environment, the variation in nutritional environment is not well characterized. Birth weight data were also not available, preventing exploration of earlier life contributions. Finally, the number of available individuals 15-y after recruitment to the original RCT constrained the sample size; therefore, a priori sample-size calculations could not be performed for these specific questions. Our data will help inform sample-size estimates for future work on this topic.

In conclusion, these findings present potential biochemical mechanisms linking greater PMWG in early life to long-term metabolic health, characterized by lower urinary muscle-related metabolites, altered lipid profiles, and higher fasting sugars, which together may contribute to cardiometabolic NCD risk in later life. These metabolites should be assessed in other cohorts to confirm associations with adult NCD, and, if validated, could serve as early markers to guide interventions. Although preventing childhood malnutrition remains the priority, elucidating these mechanisms will inform the development of novel postdischarge strategies to ensure recovery growth does not come at the expense of long-term metabolic resilience.

## Author contributions

The authors’ responsibilities were as follows – MK, AK, JRS, AC, NL: designed research; EW, AK, CD: conducted research; EW: analyzed data and had primary responsibility for final content; EW, JRS: wrote the draft manuscript; and all authors: reviewed and contributed to the final manuscript.

## Data availability

Data described in the manuscript will be made available upon request pending application and approval from https://datacompass.lshtm.ac.uk/id/eprint/2655/.

## Funding

This research was funded by the UK Medical Research Council (MRC)/Global Challenges Research Fund (GCRF) (grant number: MR/V000802/1). The follow-up 15-y postdischarge was funded by The Wellcome Trust/Clinical PhD Programme Fellowship (203919/Z/16/Z). JRS is supported by NIHR Southampton Biomedical Research Centre (NIHR203319) and the BBSRC (UKRI775; BB/W00139X/1). The supporting source was not involved in the study design; collection, analysis, and interpretation of data; writing of the report; or restrictions regarding publication.

## Conflict of interest

MK reports financial support was provided by UK Research and Innovation Medical Research Council. JRS reports financial support was provided by UK Research and Innovation Medical Research Council. AK reports financial support was provided by Wellcome Trust. If there are other authors, they declare that they have no known competing financial interests or personal relationships that could have appeared to influence the work reported in this paper.
