# Supplementary material for: Postmalnutrition weight gain is associated with changes to muscle and energy metabolism in adolescence: a cohort analysis
Source: Am J Clin Nutr. 2025 Nov 29;123(2):101130. doi: 10.1016/j.ajcnut.2025.101130 (PMC12917220; doi:10.1016/j.ajcnut.2025.101130)
Supplement: Multimedia component 2 [file mmc2.docx]

Post-malnutrition weight gain is associated with changes to muscle and energy metabolism in adolescence: a cohort analysis

Elizabeth Wimborne, Amir Kirolos, Natasha Lelijveld, Colleen Deane, Grace O’Donovan, Thandile Nkosi-Gondwe, Amelia Crampin, Marko Kerac, Jonathan Swann

**Supplementary Figures**


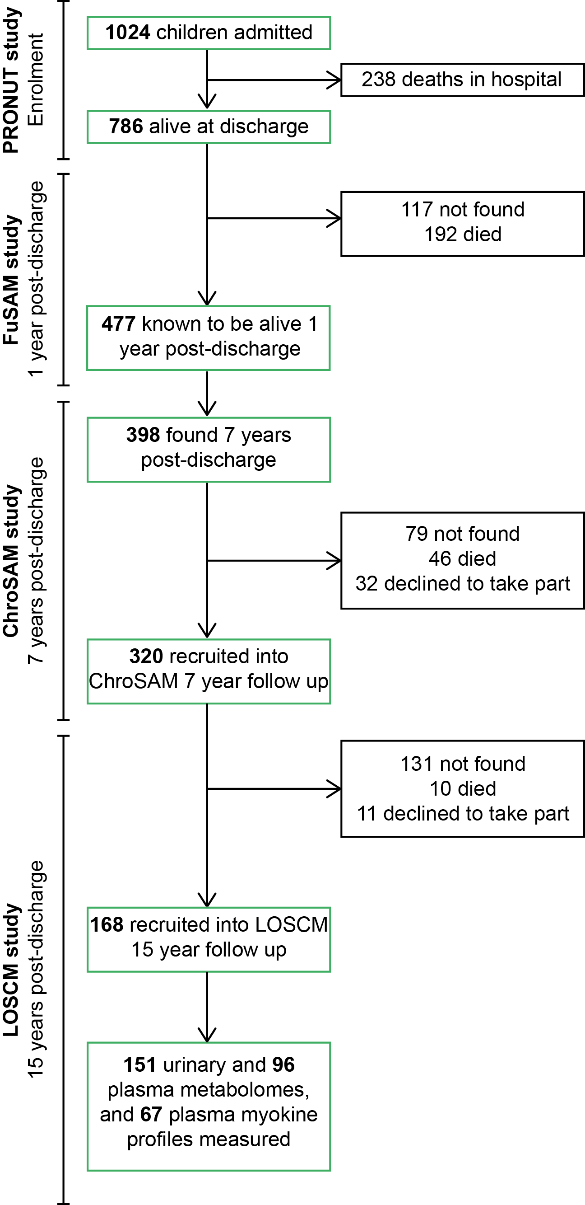


**Supplementary Figure 1**

Recruitment and follow-up of participants. Of the 168 individuals recruited at follow up, urinary metabolic profiles were obtained for 151 participants where sample volume was sufficient. Similarly, plasma metabolomics was performed in a subset of these individuals where a blood sample was provided, and sample volume was sufficient for analysis. Plasma myokines were measured subsequently for participants with adequate remaining volume. PRONUT, Probiotics and prebiotics for severe acute malnutrition; FuSAM, Follow-Up of Post-Discharge Growth and Mortality after Treatment for Severe Acute Malnutrition; ChroSAM, Chronic disease outcomes after severe acute malnutrition in Malawian children; LOSCM, Long-term outcomes after severe childhood malnutrition in adolescents in Malawi.
